# Identification of Novel Candidate Genes Associated With the Symbiotic Compatibility of Soybean With Rhizobia Under Natural Conditions

**DOI:** 10.1002/pld3.70069

**Published:** 2025-05-04

**Authors:** Masayoshi Teraishi, Kosuke Sakaguchi, Takanori Yoshikawa

**Affiliations:** ^1^ Graduate School of Agriculture Kyoto University Kyoto Japan; ^2^ National Institute of Genetics Mishima Japan

**Keywords:** quantitative trait loci (QTL) analysis, RNA‐seq, root nodule, soybean

## Abstract

A robust symbiotic relationship between soybean and rhizobia can enhance the yield and quality of soybeans by reducing nitrogen fertilizer input, thereby contributing to sustainable agriculture. However, the genetic interplay between soybean cultivars and the rhizobial species colonizing their roots under natural conditions is yet to be sufficiently assessed. In this study, we build on previous observations that have revealed a significant variation in the prevalence of rhizobial species associated with the soybean cultivars “Peking” and “Tamahomare.” Using recombinant inbred lines derived from a cross between Peking and Tamahomare, we performed quantitative trait loci (QTL) analysis of the proportion of *Rhizobium* species present in the root nodules of these cultivars and accordingly identified a major QTL on chromosome 18, accounting for 42% of the phenotypic variation, which was subsequently localized to a 240‐kb region. RNA‐seq analysis indicated that a single gene harboring nucleotide binding site–leucine‐rich repeat domains exhibited markedly different expression within the QTL region in the parent cultivars. As this locus is distinct from the chromosomal regions containing known nodule‐related genes, such as *Rj* and *rj*, we speculate that it represents a novel gene involved in the symbiosis between rhizobia and soybeans. Further research on the function and role of this new gene could potentially contribute to enhancing soybean yield, and hence sustainable agriculture, under low‐nitrogen fertilization conditions.

## 1 Introduction

Soybean ( *Glycine max* ) is among the most economically important crops worldwide that is highly suitable for human and animal consumption and a source of 30% of the world's oil derived from processed crops. Soybean originated in northern China and its cultivation has subsequently expanded worldwide, particularly in North and South America. The use of soybean in biofuel production is further increasing its economic impact, and this growing economic importance has led to increased efforts to improve soybean productivity in recent years.

Leguminous plants, including soybeans, are characterized by a unique form of symbiosis with rhizobial bacteria for facilitating the fixation of nitrogen (Doyle and Luckow 2003), which serves as a major source of nitrogen in soil (Schaedel et al. 2021). Leguminous crops are cultivated on 12%–15% of the arable land worldwide (Graham and Vance 2003), and the nitrogen fixed by these crops provides 40 million tons of nitrogen annually to agricultural land (Galloway et al. 2008). The N‐fixing capacity of legumes contributes to the nitrogen supply of both natural and arable ecosystems, and in the latter case, the effective utilization of nitrogen‐fixing capacity is necessary for developing sustainable and low‐input agriculture.

Several genetic factors, referred to as *Rj* or *rj* genes, which regulate nodulation traits, have been identified in soybeans in response to inoculation with specific rhizobial strains. However, the profiles of such genetic factors under uninoculated and natural conditions are yet to be sufficiently investigated.

In a previous study (Ramongolalaina et al. 2018), performed in a natural environment under noninoculated conditions, we identified novel genes located on chromosome 18 in soybeans that control the compatibility between rhizobial strains and host plants. However, we were unable to identify any genes responsible for nodulation in this region. In the present study, we narrowed down the quantitative trait loci (QTL) region and selected candidate genes based on RNA‐seq analysis.

## 2 Materials and Methods

### 2.1 Plant Materials

In this study, we used 113 F_6_‐derived recombinant inbred lines (RILs), derived from a cross between the parent cultivars “Tamahomare” and “Peking.” The major nodulating rhizobial species in the root nodules of Tamahomare was identified as *Bradyrhizobium japonicum* , whereas that in the root nodules of Peking was *Bradyrhizobium elkanii* .

Seeds of each soybean line, sterilized by soaking in 70% ethanol for 30 s and 2.5% sodium hypochlorite solution for 3 min, were sown in 32 cell plug trays (PEC‐32: cell size 58 mm × 58 mm, height 63 mm; Nisshin Nohkoh Co. Ltd Japan) filled with 50% sterilized vermiculite and 50% intact soil (gray lowland soil, pH 6.2, organic matter content 2.3%) derived from a soybean‐planted field of the Kyoto University experimental farm. Seedlings were grown in an incubator (CLE‐303; Tomy Co. Ltd Japan) set at 28°C under an 18/6 h photoperiod, with the order and alignment of lines being randomized, and were moisturized daily with sterile distilled water. Plants were pull out from the trays at the fourth trifoliolate stage, 4–6 weeks after sowing. Soil was removed from plants to harvest nodules formed by native rhizobial strains in the soil, with 24 nodules being randomly harvested from each line. Soil samples were ground prior to analysis.

### 2.2 DNA Extraction

The harvested nodules were initially washed to remove adhering soil and were thereafter surface‐sterilized with 70% ethanol for 1 min and 2.5% sodium hypochlorite solution for 3 min, followed by rinsing with sterilized water. Subsequently, the nodules were placed individually into tubes, submerged in 300 μL of TPS buffer containing 100‐mM Tris‐HCl, 1‐M KCl, and 10.0‐mM EDTA, and then crushed using a multi‐beads shocker (Yasui Kikai Co. Japan). Following centrifugation at 10,000 × *g* for 1 min, the DNA in the supernatant was treated with isopropanol, washed twice with 70% ethanol, and dissolved in TE buffer.

### 2.3 PCR‐Based Amplification and the Classification of Rhizobial Species

To identify the nodule‐forming rhizobial species, we performed DNA sequence analysis of the ITS region between the 16S and 23S ribosomal RNA (rRNA) genes of nodule bacteria. In our previous study (Ramongolalaina et al. 2018), rhizobial species were identified based on restriction fragment length polymorphisms of the PCR products of the ITS region between the 16S and 23S rRNA genes. In the present study, however, to identify the nodule bacteria, we performed Illumina MiSeq–based high‐throughput analysis using information of a partial sequence of the ITS region.

A conserved tRNA‐Ala sequence within the ITS region of *Bradyrhizobium* species was used for designing the forward primer for the first round of PCR, whereas the reverse primer was designed using the ITS‐R sequence at the end of the 23S rRNA gene (Akao 2004). An eight‐base index, i.e., the i5/i7 index, was inserted between the first primer and P5/P7 sequence (Figure S5). The primers used for amplicon sequencing are listed in Table S4. Twenty‐four forward and reverse primers were mixed to generate 576 primer sets containing 10.0‐μM concentrations of each primer.

For the first PCR analysis, 0.1 μL of DNA template was mixed with 10 μL of EmeraldAmp MAX PCR Master Mix (TaKaRa Bío. Co. Japan) and 0.2 μL of a primer set containing 10.0 μM of each primer, with the volume being made up to 20 μL with distilled water. The PCR cycle consisted of a pre‐denaturation at 96°C for 2 min, followed by 25 cycles of denaturation at 98°C for 20 s, annealing at 62°C for 15 s, and extension at 72°C for 1 min, with a final extension at 72°C for 1 min. The PCR products were purified using a LaboPass Gel Extraction Kit (Hokkaido System Science Co. Japan) following electrophoresis. Samples of all 576 PCR products were subsequently mixed in a single tube and subjected to amplicon sequencing using an MiSeq platform (2 × 300 bp) (Illumina, USA) conducted by Bioengineering Laboratory Co. Ltd. (Japan).

### 2.4 Identification of Rhizobial Species

Primer and index sequences were trimmed from fastq files using Cutadapt v 4.5 (Martin 2011) using the “‐‐no‐indels–discard‐untrimmed” option. Quality filtering was performed using fastp v 0.23.4 (Chen et al. 2018), and the processed fastq files were divided into RILs. The manifest file was imported into QIIME2 v 2023.9 (Bolyen et al. 2019). Classification of nodule bacteria was performed using QIIME2 v 2023.9 based on known *Bradyrhizobium* ITS sequences.

### 2.5 Construction of a Linkage Map

A linkage map of RILs was constructed using the GRAS‐Di technology (genotyping by random amplicon sequencing‐Direct) developed by TOYOTA (Enoki and Takeuchi 2018). The DNA samples of RILs were sent to Eurofins Genomics (Japan), which performed genotyping using GRAS‐Di, and the co‐dominant DNA markers accrued by GRAS‐Di were used for linkage map construction using MapMaker 3.0 (Lander et al. 1987).

The QTL analysis was performed using the composite interval mapping function of the R/qtl package and the Haley–Knott regression method. The limit of detection (LOD) significance threshold for detecting QTLs was calculated by performing 1000 iterations using the R/qtl permutation test and the correct LOD‐score were found to be greater than 3.0.

### 2.6 RNA‐Seq Analysis

For RNA‐seq analysis, total RNA was extracted from soybean roots of the parental genotypes Peking and Tamahomare using RNAiso Plus (TaKaRa Bio Co. Japan). Soybean plants were cultivated in a mixture of vermiculite and soil and were harvested after 1 month for the collection of nodules and soil. Total RNA was extracted from roots and purified using RNeasy Mini Spin Columns (Qiagen, Japan) following the manufacturer's protocols, with three biological replicates being used in each genotype. Purified RNA samples (with an RIN value greater than 7.0) were sent to Azenta Japan Corp. (Japan) and therein sequenced using the Illumina NovaSeq 2x150bp sequencing platform. The RNA‐seq data were analyzed using the RaNA‐Seq program with default parameters, with Glycine_max_v2.0 being used as a reference genome assembly (https://doi.org/10.1093/bioinformatics/btz854).

## 3 Results

### 3.1 QTL Analysis

We have previously reported a major QTL associated with the compatibility of indigenous rhizobia on chromosome 18 of soybean using 93 (F_14_‐derived) RILs derived from a cross between the Peking and Tamahomare cultivars (Ramongolalaina et al. 2018). In the present study, we developed a further set of RILs, consisting of 113 F_6_‐derived lines obtained from a cross between Peking and Tamahomare, and subjected these to high‐resolution linkage mapping using GRAS‐Di technology. The linkage map thus obtained had a total length of 2846.3 cM and contained 860 markers, with an average inter‐marker distance of 3.3 cM (Table S1).

Rhizobial strains attached to the roots were divided into two groups, i.e., *B. japonicum* and *B. elkanii* , based on amplicon sequence analysis of the partial internal transcribed spacer (ITS) region between tRNA‐Ala and the end of 23S rRNA. The results of QTL analysis in terms of the ratio of rhizobial strains revealed the presence of a single major QTL, with a LOD value greater than 3.0, associated with the compatibility of indigenous rhizobia in soil, which was located between A08389 (52,593 kbp) and A35793 (57,317 kbp) on chromosome 18, and found to be responsible for 42% of the phenotypic variation (Table 1; Figure S1). The frequency distribution of RILs for the ratio of rhizobial strains between the two homozygous genotype groups in the QTL region showed a clear bias (Figure S2), with the Peking homozygous RILs tending to show a higher frequency of *B. elkanii* , whereas the Tamahomare homozygous RILs were characterized by a higher frequency of *B. japonicum* . The highest LOD value was observed between A01978 (56,531 kbp) and A35793 (57,317 kbp) (Figure S3), which is consistent with our previous detection of QTLs in the same region near the simple sequence repeat marker Sat_064 (56,334 kbp) on chromosome 18 (Ramongolalaina et al. 2018).

**TABLE 1:** QTLs detected for the nodule ratios of *Bradyrhizobium japonicum* and *Bradyrhizobium elkanii* .

QTL name	Trait	Chromosome	Peak (cM)^a^	LOD^b^	Variance(%)^c^
qBJ_18	Ratio of *B. japonicum*	18	77.6	15.13	42.33
qBE_18	Ratio of *B. elkanii*	18	77.6	15.39	42.92

^a^ Peak (cM) denotes the peak position of the QTL from the first marker of Chr.18.

^b^ LOD denotes the maximum LOD score at the peak position for the individual QTLs.

^c^ Variance denotes the phenotypic variation explained by the QTL.

### 3.2 Fine Mapping of the Identified QTL

RILs showing recombination within the identified QTL region were selected, and to narrow down this region, we designed additional PCR‐based markers for detailed genotyping (Table S2).

**FIGURE 1:**
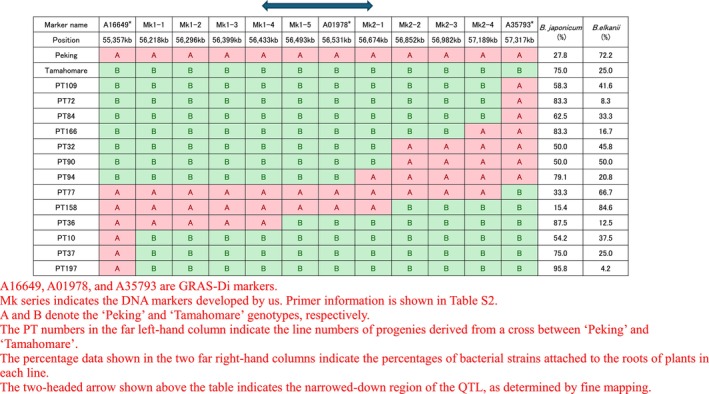
Fine mapping of the quantitative trait locus detected on Chromosome 18. A16649, A01978, and A35793 are GRAS‐Di markers. Mk series indicates the DNA markers developed by us. Primer information is shown in Table S2. A and B denote the “Peking” and “Tamahomare” genotypes, respectively. The PT numbers in the far left‐hand column indicate the line numbers of progenies derived from a cross between “Peking” and “Tamahomare.” The percentage data shown in the two far right‐hand columns indicate the percentages of bacterial strains attached to the roots of plants in each line. The two‐headed arrow shown above the table indicates the narrowed‐down region of the QTL, as determined by fine mapping.

Finally, the locations of genes determining the compatibility with rhizobia were narrowed down to a region between 56,433 and 56,675 kbp on chromosome 18 (Figure 1), and on the basis of the genome assembly sequence data of Williams 82 (Glyma.Wm82.a2), we obtained 22 predicted gene models, designated Glyma.18g280800–Glyma.18g283100 (Table 2).

**TABLE 2:** Candidate genes within the narrowed QTL region.

Marker	Gene name (Wm82.a4.v1)	Gene name (Wm82.a2.v1)	Annotations
Mk1‐4			
	Glyma18g51591	Glyma.18G280800	ROTUNDIFOLIA like 21
	Glyma18g51600	Glyma.18G280900	Allene‐oxide cyclase
	Glyma18g51626	Glyma.18G281000	Uncharacterized protein
	Glyma18g51643	Glyma.18G281100	DNA‐directed RNA polymerase I and III subunit Rpc19
	Glyma18g51660	Glyma.18G281200	60S ribosomal protein L30‐like
	Glyma18g51670	Glyma.18G281300	Potential peptide *N*‐acetyl transferase
	Glyma18g51680	Glyma.18G281400	Ethylene‐responsive transcription factor
Mk1‐5			
		Glyma.18G281451	MEI2‐like protein 5
	Glyma18g51715	Glyma.18G281500	Disease resistance protein
	Glyma18g51741	Glyma.18G281600	NB‐ARC domain disease resistance protein
A01978			
	Glyma18g51765	Glyma.18G281700	NB‐ARC domain disease resistance
	Glyma18g51790	Glyma.18G281800	myb/SANT‐like DNA‐binding domain protein
	Glyma18g51810	Glyma.18G282000	ADP‐ribosylation factor GTPase‐activating
	Glyma18g51820	Glyma.18G282100	Protein kinase superfamily protein
	Glyma18g51830	Glyma.18G282200	Serine carboxypeptidase‐like
	Glyma18g51840	Glyma.18G282300	C‐terminal domain phosphatase‐like
	Glyma18g51850	Glyma.18G282400	Protein bicaudal C homolog 1‐B‐like
	Glyma18g51871	Glyma.18G282500	E3 ubiquitin protein ligase DRIP2‐like
	Glyma18g51880	Glyma.18G282600	Disease resistance‐responsive
	Glyma18g51890	Glyma.18G282700	Histone‐lysine *N*‐methyltransferase SUVR5‐like isoform X2
	Glyma18g51900	Glyma.18G282800	Ran binding protein
		Glyma.18G282900	Unknown protein
	Glyma18g51911	Glyma.18G283000	RNA recognition motif
	Glyma18g51920	Glyma.18G283100	Eukaryotic aspartyl protease family
Mk2‐1			

*Note:* Information regarding gene names and annotations was derived from the Soybase database. Detailed marker information is presented in Figure 1 and Table S2.

### 3.3 RNA‐Seq Analysis and Candidate Genes

To identify the potential candidate genes responsible for QTL, we performed RNA‐seq analysis using RNA derived from roots of the Peking and Tamahomare cultivars 1 month post‐germination. We accordingly identified a total of 662 differentially expressed genes (DEGs) (with a greater than two‐fold change) between Peking and Tamahomare, of which 265 and 397 were significantly expressed in Peking and Tamahomare, respectively. Notably, the results of RNA‐seq analysis revealed that only a single gene within the QTL region, Glyma.18g281700, was differentially expressed between the parents with a *p*_adj_ < 0.05 and fold change > 2 (Figure S4; Table S3), showing significantly higher expression in Peking than in Tamahomare. Glyma.18g281700 was established to encode NBS–ARC (apoptosis, R proteins, and CED‐4) and LRR domains that are often observed in disease resistance (R) genes (McHale et al. 2006).

## 4 Discussion

To date, a number of genes regulating nodulation, referred to as *Rj*(s) and *rj*(s) genes, have been reported in soybeans, of which *Rj2*, *Rj3*, *Rj4*, and *Rfg1* have been identified as dominant alleles that restrict nodulation in specific strains (Hayashi et al. 2012). *Rj2* and *Rfg1* are allelic genes located on chromosome 16 and encode a member of the Toll/interleukin‐1 receptor–NBS–LRR resistance protein (Fan et al. 2017), whereas the *Rj4* allele is located on chromosome 1 and encodes a thaumatin‐like protein (Hayashi et al. 2014; Tang et al. 2014, 2016) and *Rj3* appears to be associated exclusively with nodulation by *B. elkanii* USDA33 (Vest 1970) but has not yet been identified. Other recessive genes that regulate nodulation induce nonnodulation (*rj1*, *rj5*, and *rj6*) (Pracht et al. 1993; Lee et al. 2011; Indrasumunar et al. 2011) and hypernodulation (*rj7*) (Nishimura , Hayashi, et al. 2002; Nishimura , Ohmori, et al. 2002) phenotypes.

Ramongolalaina et al. (2018) demonstrated, for the first time, the chromosomal location of genes responsible for the proportion of nodulating bacterial strains in roots under natural, noninoculated conditions. In the present study, we successfully narrowed down the location of the gene underlying the identified QTL to a 240‐kb interval, wherein 22 gene models were predicted. RNA‐seq revealed a single gene within the QTL interval that was significantly differentially expressed between Peking and Tamahomar.

Three consecutive genes, namely, Glyma.18g281500, Glyma.18g281600, and Glyma.18g281700, have ubiquitin‐like protease 1, NBS–ARC, and LRR domains, respectively (Jebanathirajah et al. 2002; Steele et al. 2019; Wei et al. 2023) and show more than 90% sequence similarity. However, although Glyma.18g281500 was located just outside the QTL region and Glyma.18g281600 was located within the QTL region, neither of these two genes showed differential expression between the two parents.

The genes *Rpp1* and *Rpp1b* that confer resistance to *Phakopsora pachyrhizi*, the causative agent of rust, are located within the same region on chromosome 18 (Pedley et al. 2019; Barros et al. 2023; Wei et al. 2023; Yamanaka et al. 2023). Pedley et al. (2019) have reported that eight genes homologous to the NBS–LRR family of disease R genes were found in four bacterial artificial chromosome contigs on chromosome 18, including the *Rpp1* locus. The aforementioned Glyma.18g281500, 281600, and 281700 genes were accordingly considered candidates for *Rpp1*, among which Glyma.18g281600 was the most highly expressed in PI200492. However, Barros et al. (2023) showed that the *Rpp1* gene in PI594756 was located immediately upstream of *Rpp1* in PI200492, and suggested that genomic variations, such as presence/absence and copy number variations, might contribute to the diversification of disease resistance genes.

In conclusion, we detected a single major QTL located on chromosome 18 associated with the symbiotic compatibility of soybean–rhizobia nodules, although no genes known to be implicated in the symbiosis between rhizobia and soybeans were identified on chromosome 18 (Table S5). A single candidate gene was identified showing differential transcription between Peking and Tamahomare, which harbors an NBS–LRR domain and has been reported as a candidate for the soybean rust resistance–associated gene *Rpp1*.

## Author Contributions

MT conceived and designed the study. MT and TY developed the mapping population, constructed the genetic linkage map, and performed the fine mapping. MT and KS collected the phenotypic data. MT performed RNA‐seq analysis and wrote the manuscript. All the authors contributed to the manuscript and approved the submitted version.

## Conflicts of Interest

The authors declare no conflicts of interest.

## Supporting information


**Data S1** Supporting Information

**Figure S1** QTL location on Chromosome 18
**Figure S2.** Distribution of the recombinant inbred lines (RILs) for the ratio of rhizobial strains used in homozygous genotypes at the QTL region from the RILs derived from ‘Peking’ and ‘Tamahomare’
**Figure S3.** Schematic view of the location of molecular markers in the vicinity of the QTL region on Chromosome 18
**Figure S4.** Heatmap of the 22 candidate genes in ‘Peking’ and ‘Tamahomare’.The green and red blocks represent overexpressed and underexpressed genes, respectively. P1–P3 indicate the replicates of ‘Peking’, and T1–T3 indicated those of ‘Tamahomare’
**Figure S5.** Schematic representation of PCR amplification for the ITS region. The ITS region, amplified using the ITS‐F and ITS‐R primers, is suitable for PCR‐RFLP and Sanger sequencing. However, as the amplified ITS region is approx. 900 bp long, it is not suitable for shotgun amplicon sequencing. Therefore, primers were designed from the tRNA‐Ala site.

**Table S1** Genetic map summary generated using GRAS‐Di
**Table S2** List of primer sequences used for fine mapping of the QTL located on Chromosome 18
**Table S3** Summary of differentially expressed genes within the QTL region
**Table S4** Primers used for amplicon sequencing
Table S5: GWAS QTLs located in the QTL region on chromosome 18

## Data Availability

The RNA‐seq raw data used in this study were downloaded from the DRA database in the DNA Data Bank of Japan under the BioProject accession number PRJDB17790.
