# Correction to “Identification of Novel Candidate Genes Associated With the Symbiotic Compatibility of Soybean With Rhizobia Under Natural Conditions”

**DOI:** 10.1002/pld3.70083

**Published:** 2025-05-23

Teraishi, M. , Sakaguchi, K. and Yoshikawa, T. (2025), Plant Direct, 9: e70069. 10.1002/pld3.70069.40330701 PMC12050213

In Funding, “This work was supported by MEXT|Japan Society for the Promotion of Science (JSPS) (22K05571) and the Kyoto University Foundation (KUF).” was incorrect.

This should have read: “This work was supported by MEXT|Japan Society for the Promotion of Science (JSPS) (22K05571 and 24KK01695) and the Kyoto University Foundation (KUF).”

We apologize for this error.

