# Supplementary material for: Identification of Novel Candidate Genes Associated With the Symbiotic Compatibility of Soybean With Rhizobia Under Natural Conditions
Source: Plant Direct. 2025 May 4;9(5):e70069. doi: 10.1002/pld3.70069 (PMC12050213; doi:10.1002/pld3.70069)
Supplement: Supplementary file 2 — Figure S1 QTL location on Chromosome 18 Figure S2. Distribution of the recombinant inbred lines (RILs) for the ratio of rhizobial strains used in homozygous genotypes at the QTL region from the RILs derived from ‘Peking’ and ‘Tamahomare’ Figure S3. Schematic view of the location of molecular markers in the vicinity of the QTL region on Chromosome 18 Figure S4. Heatmap of the 22 candidate genes in ‘Peking’ and ‘Tamahomare’.The green and red blocks represent overexpressed and underexpressed genes, respectively. P1–P3 indicate the replicates of ‘Peking’, and T1–T3 indicated those of ‘Tamahomare’ Figure S5. Schematic representation of PCR amplification for the ITS region. The ITS region, amplified using the ITS‐F and ITS‐R primers, is suitable for PCR‐RFLP and Sanger sequencing. However, as the amplified ITS region is approx. 900 bp long, it is not suitable for shotgun amplicon sequencing. Therefore, primers were designed from the tRNA‐Ala site. [file PLD3-9-e70069-s003.pptx]

## Slide 1
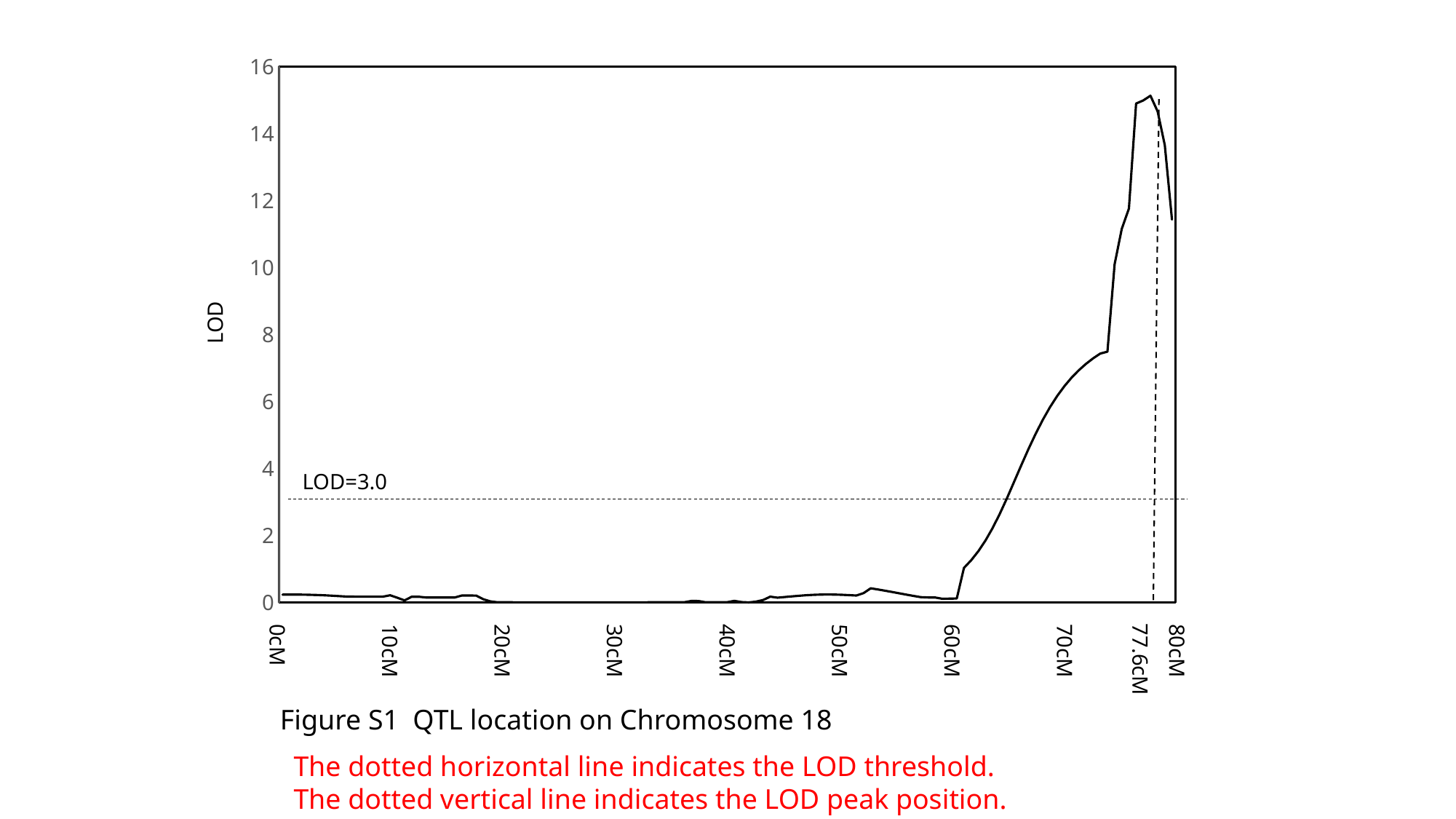

### Chart
| Category | |
|---|---|
| 1.01 | 0.23239857741986336 |
| 2.0099999999999998 | 0.23455094087217584 |
| 3.01 | 0.23449165967539606 |
| 4.01 | 0.23203355290782365 |
| 5.01 | 0.2271251566733531 |
| 6.01 | 0.2198691816169545 |
| 7.01 | 0.21052577013328796 |
| 8.01 | 0.1994909987898492 |
| 9.01 | 0.1872523631425746 |
| 10.01 | 0.17433101656974812 |
| 10.14 | 0.17280859726343625 |
| 10.18 | 0.17280859726343625 |
| 10.220000000000001 | 0.17280859726343625 |
| 10.26 | 0.17280859726343625 |
| 10.299999999999999 | 0.17280859726343625 |
| 10.34 | 0.21400446893333303 |
| 11.110000000000001 | 0.14095613707720606 |
| 11.87 | 0.060454009028173605 |
| 12.870000000000001 | 0.1690843049338749 |
| 12.889999999999999 | 0.17037785104822376 |
| 13.389999999999999 | 0.1468432159266456 |
| 13.639999999999999 | 0.1468432159266456 |
| 13.68 | 0.1468432159266456 |
| 13.719999999999999 | 0.1468432159266456 |
| 13.969999999999999 | 0.1468432159266456 |
| 14.35 | 0.20544409040053802 |
| 14.729999999999999 | 0.20544409040053802 |
| 15.229999999999999 | 0.2043164447782762 |
| 16.23 | 0.09469270024522122 |
| 17.03 | 0.03010637922173817 |
| 18.029999999999998 | 0.0048777784734963725 |
| 18.05 | 0.00473185552757688 |
| 18.09 | 0.00470775218383125 |
| 19.09 | 0.0025362797743149904 |
| 19.63 | 0.0016290386016190974 |
| 20.39 | 0.0016290386016190974 |
| 20.64 | 0.0016290386016190974 |
| 20.68 | 0.0016290386016190974 |
| 20.72 | 0.0016290386016190974 |
| 20.76 | 0.0016290386016190974 |
| 20.8 | 0.0016290386016190974 |
| 20.84 | 0.0016290386016190974 |
| 20.880000000000003 | 0.0016290386016190974 |
| 20.919999999999998 | 0.0016290386016190974 |
| 20.96 | 0.0016290386016190974 |
| 21 | 0.0016290386016190974 |
| 21.04 | 0.0016290386016190974 |
| 21.08 | 0.0016290386016190974 |
| 21.34 | 0.0016290386016190974 |
| 21.6 | 0.0016290386016190974 |
| 21.64 | 0.0015200306866613813 |
| 22.41 | 0.00453142862417853 |
| 22.67 | 0.00453164577141948 |
| 22.71 | 0.00453164577141948 |
| 22.75 | 0.00453164577141948 |
| 22.79 | 0.00453142862417853 |
| 23.05 | 0.005314678722291044 |
| 23.31 | 0.04405396365530206 |
| 23.35 | 0.0419098517981457 |
| 23.61 | 0.00453164577141948 |
| 23.65 | 0.00453164577141948 |
| 23.69 | 0.004565086446526031 |
| 24.69 | 0.008541269575591252 |
| 25.259999999999998 | 0.04712637996752661 |
| 26.26 | 0.01029690501868515 |
| 27.26 | 0.0007270089627060435 |
| 28.26 | 0.024153939052772203 |
| 29.26 | 0.07310457299153342 |
| 29.59 | 0.1723886344994358 |
| 30.4 | 0.1424516301256399 |
| 31.4 | 0.16120576873766804 |
| 32.4 | 0.1804287282429107 |
| 33.4 | 0.19883456268045244 |
| 34.4 | 0.21489042967641564 |
| 35.4 | 0.22714426563055684 |
| 36.4 | 0.23457070127110247 |
| 37.4 | 0.23679363757672425 |
| 38.4 | 0.2341094805313212 |
| 39.4 | 0.2273416524725819 |
| 40.400000000000006 | 0.21759934150728716 |
| 41.4 | 0.20602843362593884 |
| 41.74 | 0.2779122048288422 |
| 41.78 | 0.42103438563454076 |
| 42.78 | 0.3869246798786184 |
| 43.78 | 0.3501114909728483 |
| 44.78 | 0.3113120562540937 |
| 45.78 | 0.2714685776008437 |
| 46.78 | 0.23169350032849342 |
| 47.78 | 0.19317266551987972 |
| 48.78 | 0.1570352388279511 |
| 49.220000000000006 | 0.14761778013512003 |
| 49.26 | 0.1470851179530657 |
| 50.019999999999996 | 0.10812151992015166 |
| 51.019999999999996 | 0.11378667428933863 |
| 52.019999999999996 | 0.11751769818336948 |
| 52.93 | 1.031023568780717 |
| 53.93 | 1.2569487698005712 |
| 54.93 | 1.527649949051892 |
| 55.93 | 1.8487514372225666 |
| 56.93 | 2.223239660317404 |
| 57.930000000000007 | 2.6486035276420377 |
| 58.930000000000007 | 3.114621049975913 |
| 59.930000000000007 | 3.604434001267749 |
| 60.929999999999993 | 4.098876966031155 |
| 61.929999999999993 | 4.580531478333198 |
| 62.93 | 5.0355363783422105 |
| 63.93 | 5.454095068282662 |
| 64.929999999999993 | 5.830668556332357 |
| 65.930000000000007 | 6.16377893836938 |
| 66.930000000000007 | 6.4551616551434154 |
| 67.930000000000007 | 6.708521021437857 |
| 68.930000000000007 | 6.9283773913675955 |
| 69.930000000000007 | 7.119291074139856 |
| 70.930000000000007 | 7.285457137302582 |
| 71.930000000000007 | 7.430557095178868 |
| 72.009999999999991 | 7.486865111935551 |
| 73.009999999999991 | 10.096036220651461 |
| 74.009999999999991 | 11.151031759097032 |
| 74.709999999999994 | 11.759173019222711 |
| 75.709999999999994 | 14.89395597337374 |
| 76.570000000000007 | 14.987207033086282 |
| 77.569999999999993 | 15.128230920102666 |
| 78.569999999999993 | 14.658161430252633 |
| 79.569999999999993 | 13.650503121745555 |
| 80.569999999999993 | 11.430549550625472 |LOD
LOD=3.0
0cM
10cM
20cM
30cM
40cM
50cM
60cM
70cM
80cM
77.6cM
Figure S1 QTL location on Chromosome 18
The dotted horizontal line indicates the LOD threshold.
The dotted vertical line indicates the LOD peak position.

## Slide 2
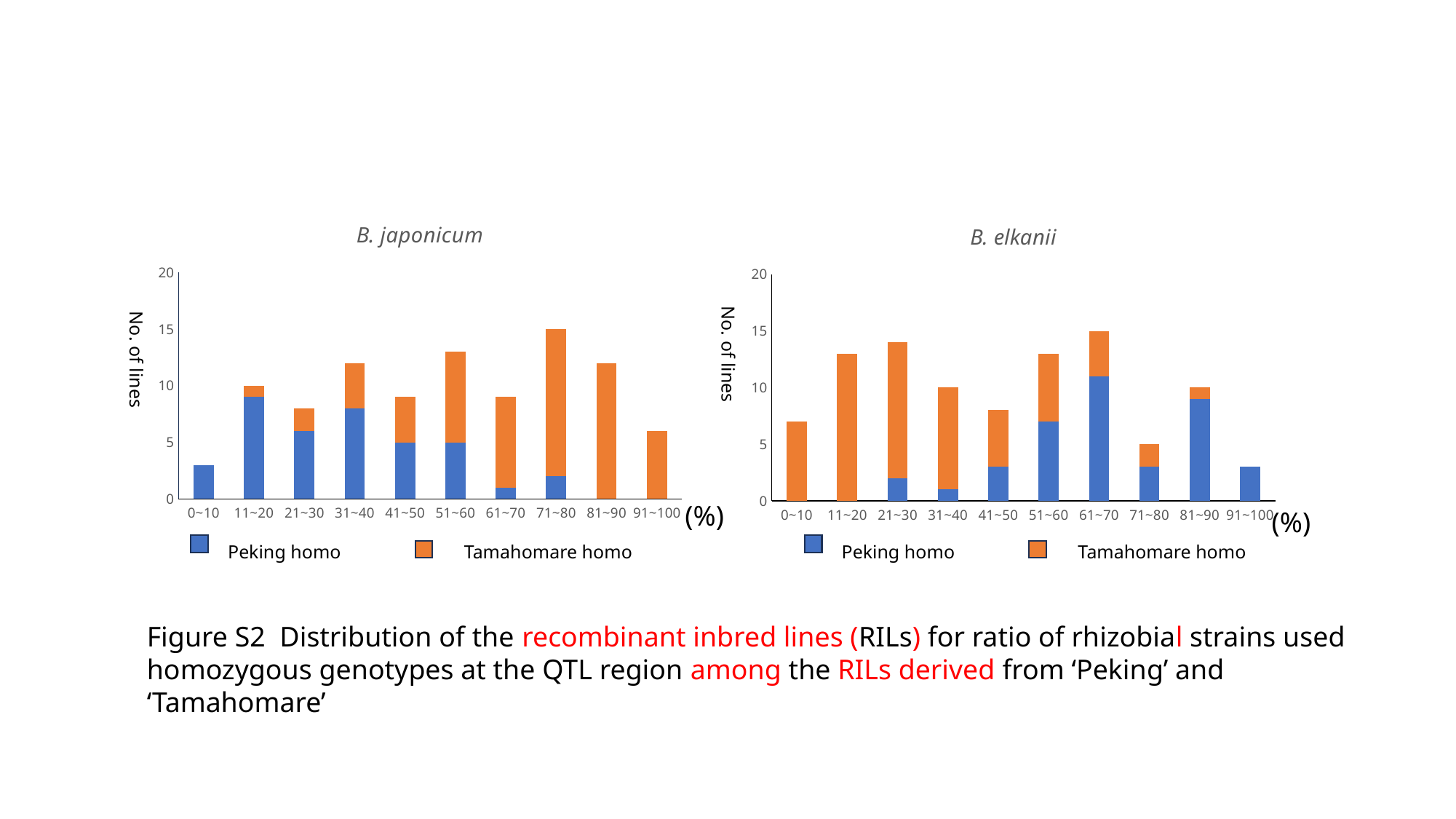

### Chart: B. japonicum
| Category | Pekingホモ型 | タマホマレホモ型 |
|---|---|---|
| 0~10 | 3.0 | 0.0 |
| 11~20 | 9.0 | 1.0 |
| 21~30 | 6.0 | 2.0 |
| 31~40 | 8.0 | 4.0 |
| 41~50 | 5.0 | 4.0 |
| 51~60 | 5.0 | 8.0 |
| 61~70 | 1.0 | 8.0 |
| 71~80 | 2.0 | 13.0 |
| 81~90 | 0.0 | 12.0 |
| 91~100 | 0.0 | 6.0 |
### Chart: B. elkanii
| Category | Pekingホモ型 | タマホマレホモ型 |
|---|---|---|
| 0~10 | 0.0 | 7.0 |
| 11~20 | 0.0 | 13.0 |
| 21~30 | 2.0 | 12.0 |
| 31~40 | 1.0 | 9.0 |
| 41~50 | 3.0 | 5.0 |
| 51~60 | 7.0 | 6.0 |
| 61~70 | 11.0 | 4.0 |
| 71~80 | 3.0 | 2.0 |
| 81~90 | 9.0 | 1.0 |
| 91~100 | 3.0 | 0.0 |No. of lines
No. of lines
(%)
(%)
Peking homo
Tamahomare homo
Peking homo
Tamahomare homo
Figure S2 Distribution of the recombinant inbred lines (RILs) for ratio of rhizobial strains used homozygous genotypes at the QTL region among the RILs derived from ‘Peking’ and ‘Tamahomare’

## Slide 3
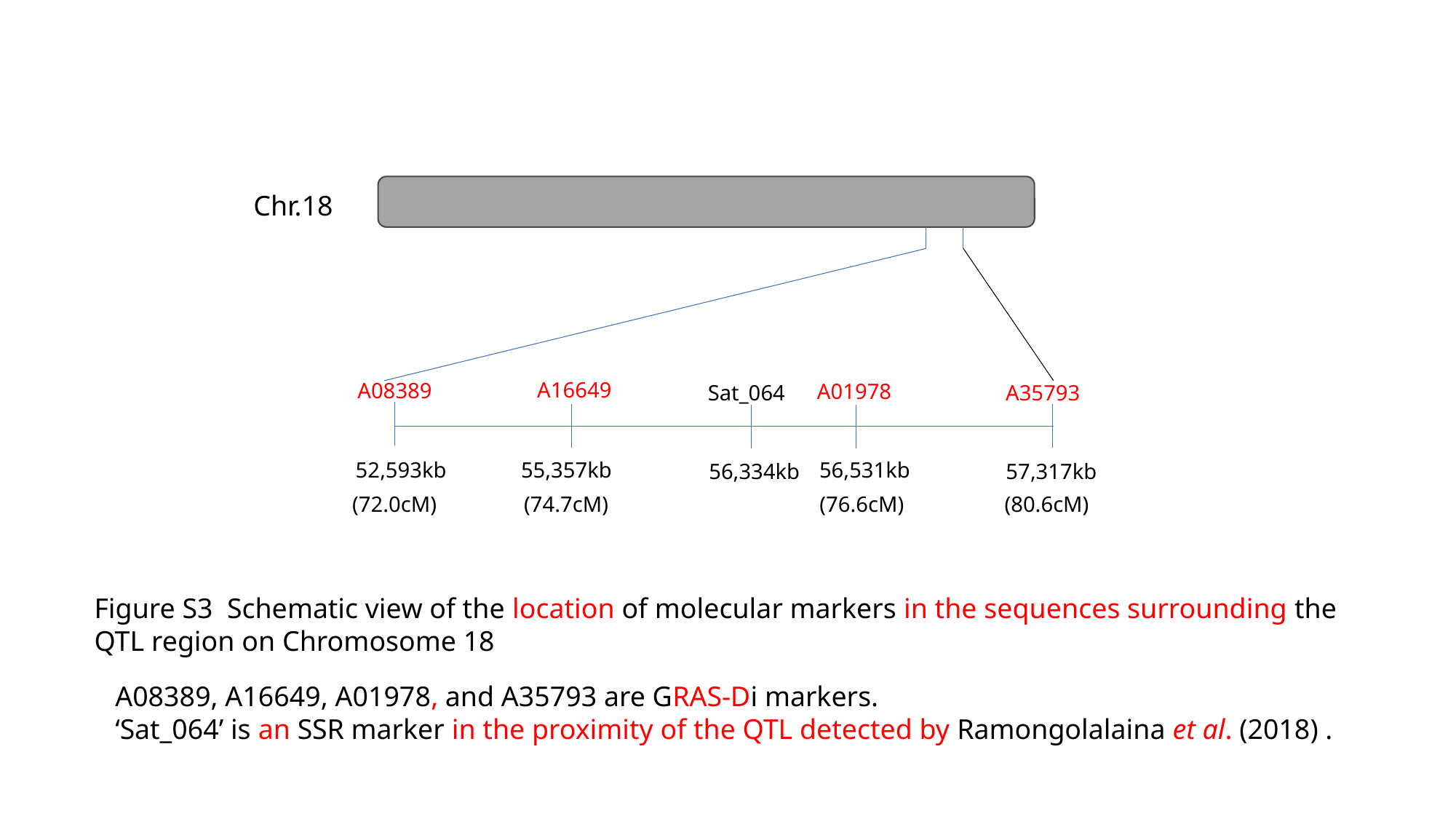

Chr.18
A16649
A08389
A01978
Sat_064
A35793
55,357kb
52,593kb
56,531kb
56,334kb
57,317kb
(72.0cM)
(74.7cM)
(76.6cM)
(80.6cM)
Figure S3 Schematic view of the location of molecular markers in the sequences surrounding the QTL region on Chromosome 18
A08389, A16649, A01978, and A35793 are GRAS-Di markers.
‘Sat_064’ is an SSR marker in the proximity of the QTL detected by Ramongolalaina et al. (2018) .

## Slide 4
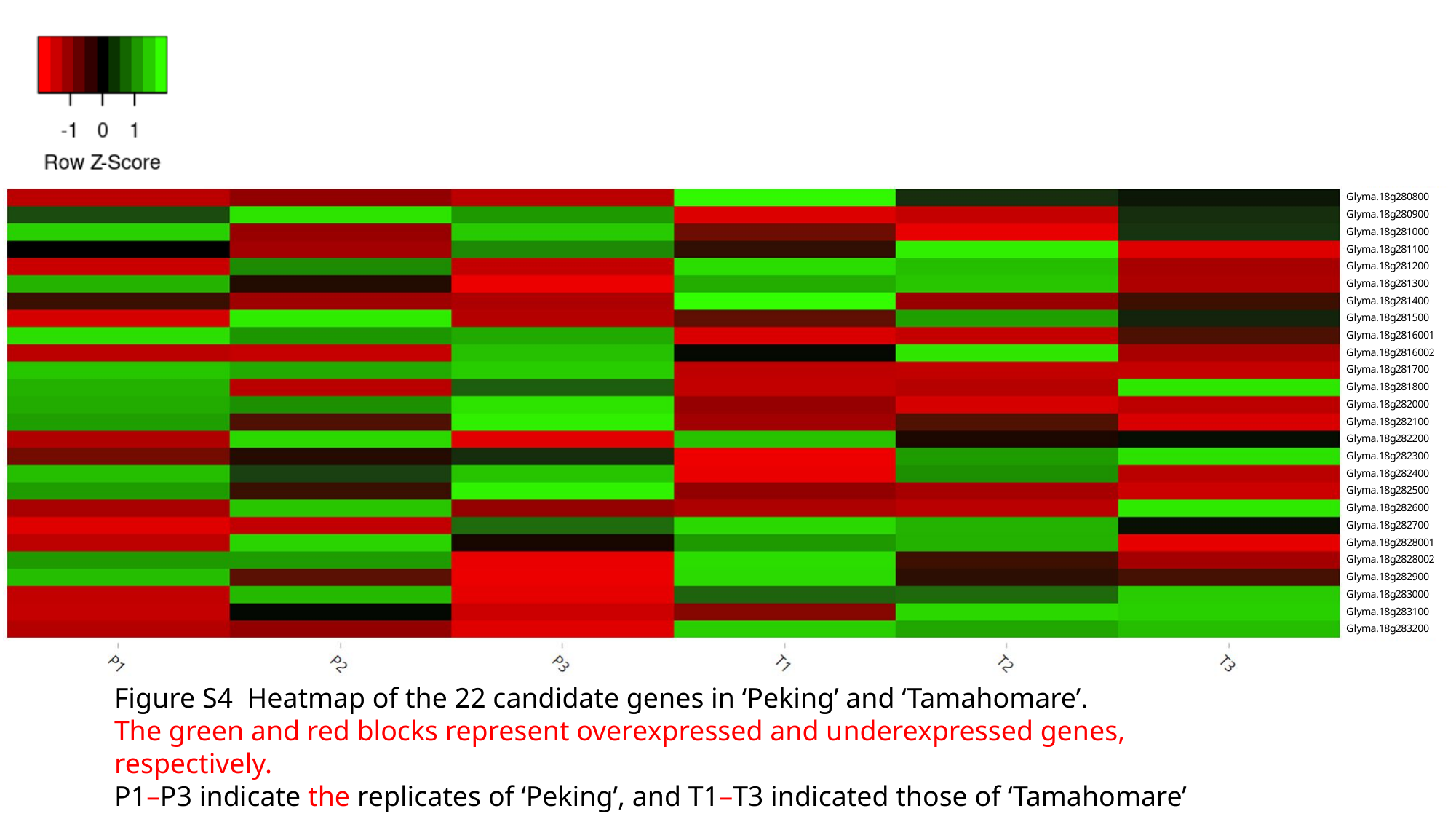

Figure S4 Heatmap of the 22 candidate genes in ‘Peking’ and ‘Tamahomare’.
The green and red blocks represent overexpressed and underexpressed genes, respectively.
P1–P3 indicate the replicates of ‘Peking’, and T1–T3 indicated those of ‘Tamahomare’

## Slide 5
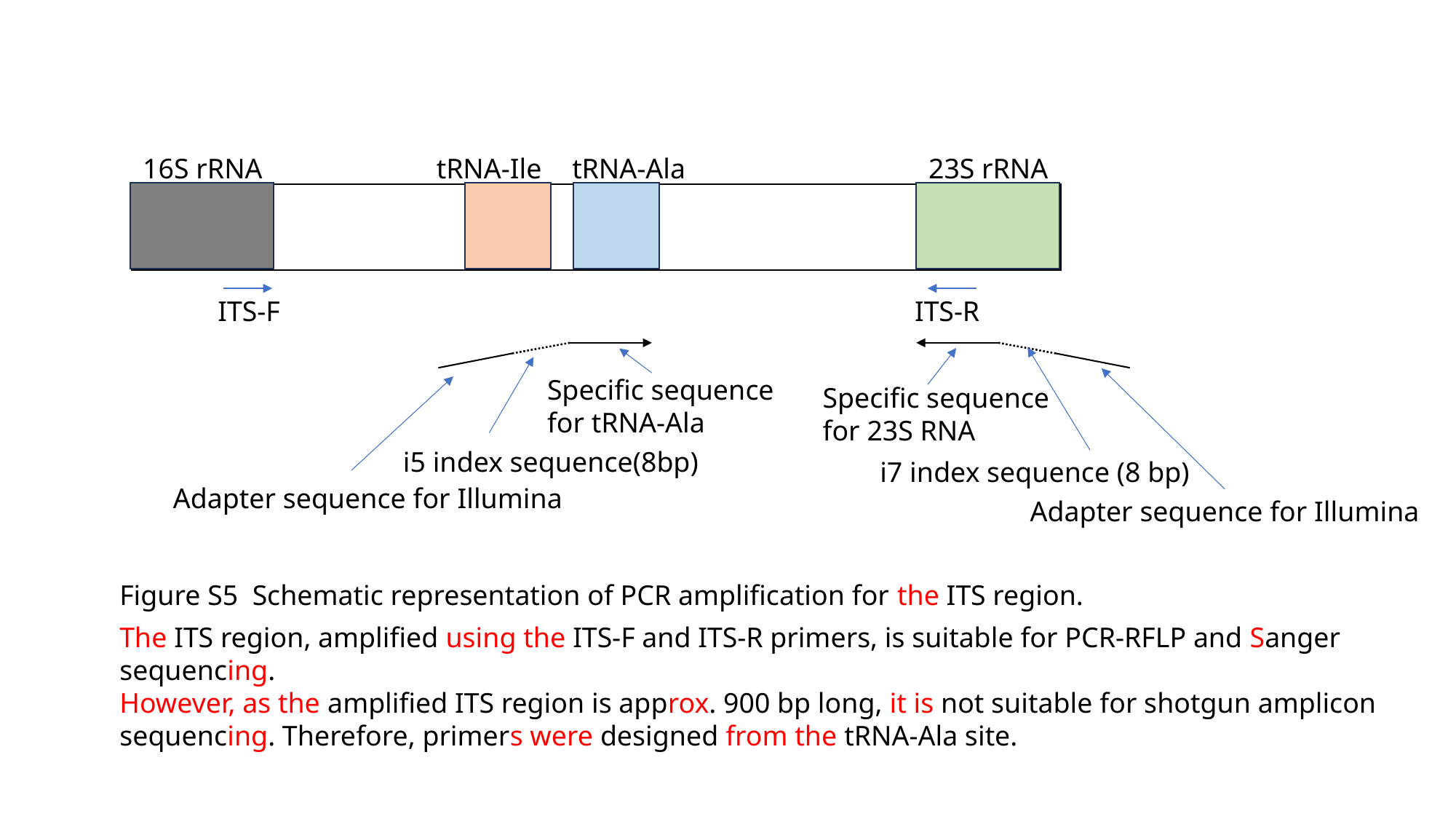

16S rRNA
tRNA-Ile
tRNA-Ala
23S rRNA
ITS-F
ITS-R
Specific sequence
for tRNA-Ala
Specific sequence
for 23S RNA
i5 index sequence(8bp)
i7 index sequence (8 bp)
Adapter sequence for Illumina
Adapter sequence for Illumina
Figure S5 Schematic representation of PCR amplification for the ITS region.
The ITS region, amplified using the ITS-F and ITS-R primers, is suitable for PCR-RFLP and Sanger sequencing.
However, as the amplified ITS region is approx. 900 bp long, it is not suitable for shotgun amplicon sequencing. Therefore, primers were designed from the tRNA-Ala site.
