# Supplementary material for: Identification of Novel Candidate Genes Associated With the Symbiotic Compatibility of Soybean With Rhizobia Under Natural Conditions
Source: Plant Direct. 2025 May 4;9(5):e70069. doi: 10.1002/pld3.70069 (PMC12050213; doi:10.1002/pld3.70069)
Supplement: Supplementary file 3 — Table S1 Genetic map summary generated using GRAS‐Di Table S2 List of primer sequences used for fine mapping of the QTL located on Chromosome 18 Table S3 Summary of differentially expressed genes within the QTL region Table S4 Primers used for amplicon sequencing Table S5: GWAS QTLs located in the QTL region on chromosome 18 [file PLD3-9-e70069-s001.docx]

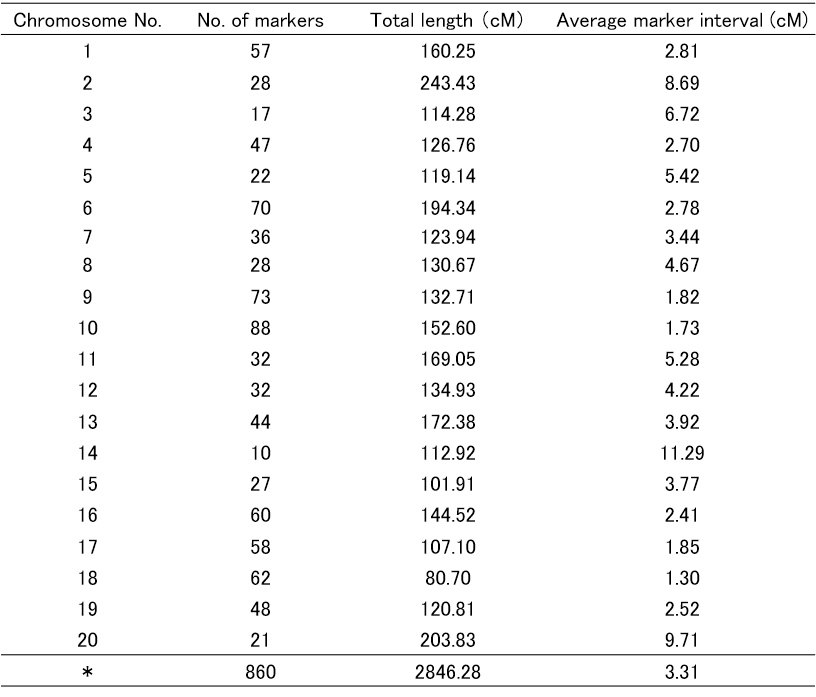


Table S1 Genetic map summary generated using GRAS-Di

The genetic map was constructed using MapMaker/EXP 3.0 (Lander *et al*., 1897).


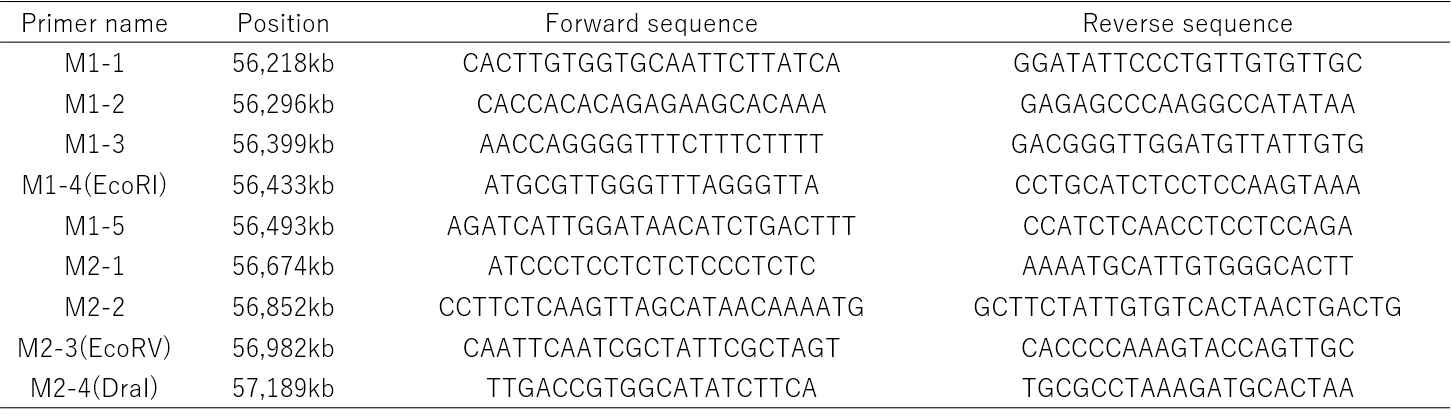


M1-4, M2-3, and M2-4 are cleaved amplified polymorphic sequence (CAPS) markers.

Other markers are indel markers.

Table S2 List of the sequence of primers used for fine mapping of the QTL located on Chromosome 18


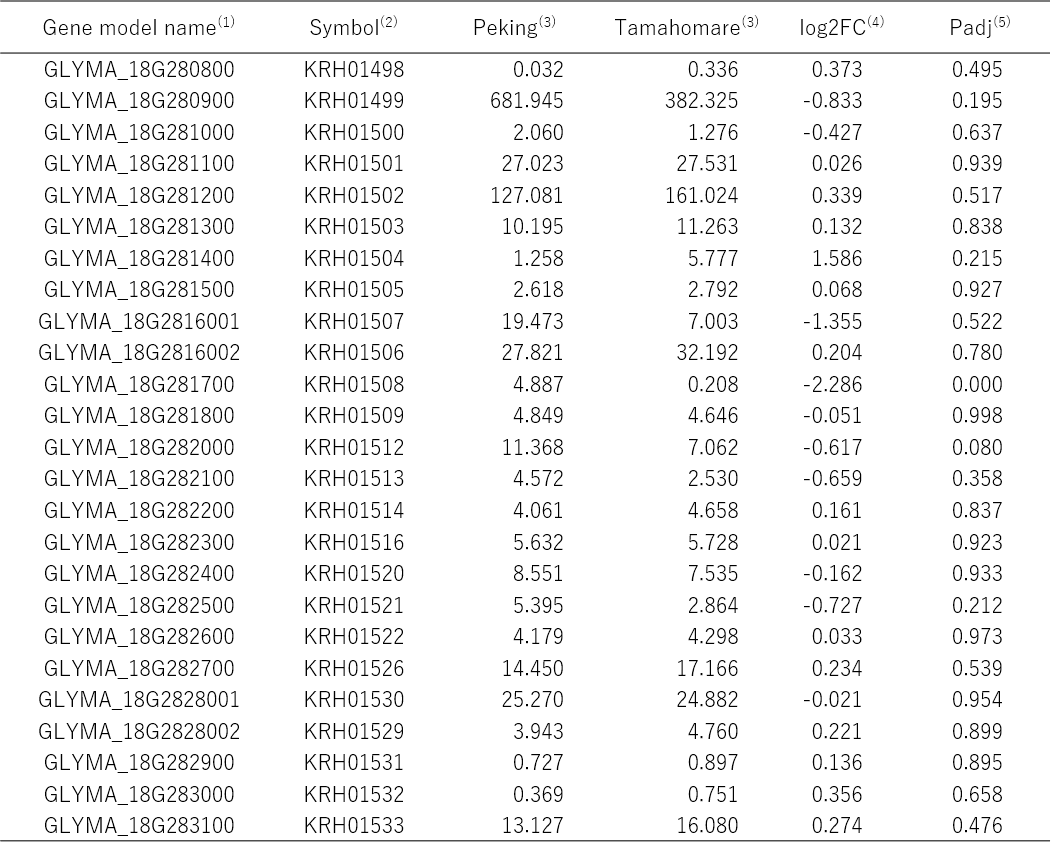


(1) Gene model names are derived from the Soybase database (https://www.soybase.org)

(2) GenBank ID, (3) the average value of three replicates, (4) Log 2-fold change, and

(5) adjusted p-values from the Wald test using the Benhamini and Hochberg method.

Table S3 Summary of the differentially expressed genes within the QTL region

1. Gene model names are derived from the Soybase database (https://www.soybase.org)
2. GenBank ID, (3) the average value of three replicates, (4) Log 2-fold change, and

(5) adjusted p-values from the Wald test using the Benhamini and Hochberg method.


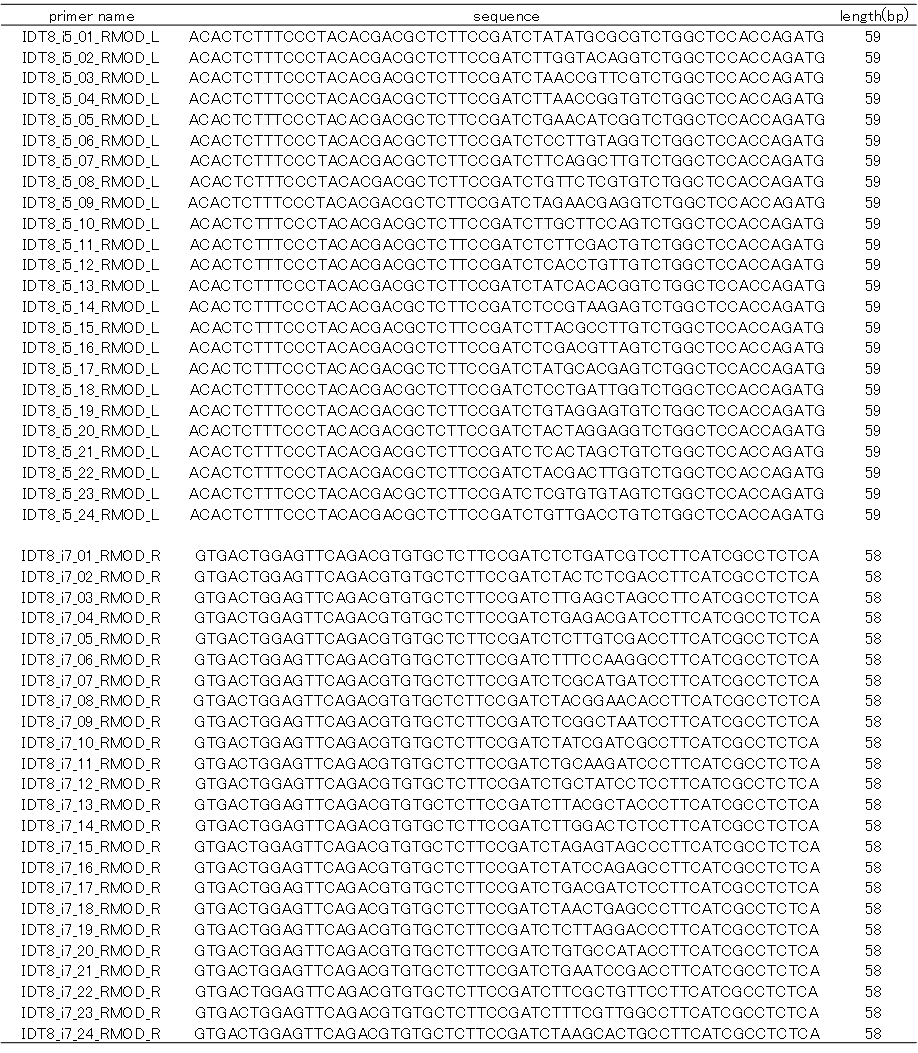


Table S4 Primers used for amplicon sequencing

Data originated from soybase (https://www.soybase.org).

Table S5 GWAS QTLs locating the QTL region on chromosome 18
