# Supplementary material for: Identification of Novel Candidate Genes Associated With the Symbiotic Compatibility of Soybean With Rhizobia Under Natural Conditions
Source: Plant Direct. 2025 May 4;9(5):e70069. doi: 10.1002/pld3.70069 (PMC12050213; doi:10.1002/pld3.70069)
Supplement: Supplementary file 1 — Data S1 Supporting Information [file PLD3-9-e70069-s002.pdf]

Plant Direct
Open Access

Identification of novel candidate genes associated with the symbiotic compatibility of soybean with rhizobia under natural conditions

Masayoshi Teraishi[1], Kosuke Sakaguchi[1], Takanori Yoshikawa[2]

Decision Letter Round 1:

July 24, 2024

Prof. Masayoshi Teraishi
kyoto university
agriculture
Kitashirakawa Oiwakecho, Sakyo-ku, Kyoto
Kyoto, N/A 606-8502
Japan

RE: Identification of novel candidate genes associated with the symbiotic compatibility of soybean with rhizobia under natural conditions

Dear Dr. Teraishi:

Thank you for submitting to Plant Direct. All required reviews have been returned and we have now finished our evaluation of your manuscript. We apologize for the delay in the process, it has been particularly challenging to have peer-reviewers for your study. In light of the reviewers' and editor's comments, further revisions are needed before the paper can be accepted for publication in Plant Direct.

Please view the editors' and reviewers' comments below and use their suggestions as a guide while you work on your revision.

When uploading the revised version of this article, please be sure to include the following:

- A word document that contains your response to the reviewers. You should respond to each

reviewer comment and note the changes made to the manuscript. If you do not agree with a reviewer's comment and choose not to make a suggested revision, please explain why. Please try to provide as complete an answer as possible to each reviewer's criticisms in the "response to reviewers" section.
- A tracked changes document
- A clean version of the latest version of the manuscript

To upload your revision, please click the link below.
https://plantdirect.msubmit.net/cgi-bin/main.plex?el=A5Lr6hp2A6oiK5I4A9ftduSdFMS5tyFQGJZoDVsMXgZ

In order to provide as timely a service as possible, we ask that your revision is resubmitted within three months after receipt of this request. If an extension is needed, please send a request, along with a brief explanation, to the editorial office at plantdirect@wiley.com .

Thank you very much for giving us an opportunity to review your work. I look forward to receiving the next version.

Sincerely,
Jonathan Fresnedo-Ramírez
Academic Editor

Gustavo Macintosh
Supervising Editor

-- Editor comments:
Please, pay particular attention to the main concerns from Reviewer 2. Plant Direct emphasizes sound scientific studies and ensure the replication of the studies is paramount. qRT-PCR validation would be highly desirable to support the soundness of your study and results.

-- Reviewer comments:
Reviewer #1:

Line 70 - Briefly mention the predominant soil type or any characterization available of the location where the soil was sampled. Example: (Ultisol, Ph 4.7, OM 2.5%)

Line 71- Was the soil ground after collection? If, yes add it to the description.

Line 97 - Redundant statement, perhaps use "Twenty-four forward and reverse primers were mixed"

Line 110 to 115 - Include the version of the software used. Include what range parameter was considered good quality for the final fastq files.

Line 137 - restructure to avoid starting the sentence with a reference. Perhaps used "Previous reports"

Line 118- Expand GRAS-Di technologies and remove them from Line 142

Line 143- "cM" Centimorgans, verify accordingly through text

Line 178- Add references (Rps genes could work)

Line 240 to 246- Different font detected, verify accordingly

Line 249- Merge with paragraph, space detected.

Table 1 - (3) phenotypic variation symbol missing within the final column of the figure.

Figure S1- Add to the description. Example: software from where the figure was generated.

Figure S4 - Blurry axis for the genes. Also, there might be image stretching, Make sure it's at least 300DPI.

Table S3- (5) P-value adjusted based on? Bonferroni?

Overall suggestion #1- It might be a good idea to include a simple table with colocalizing QTLs to the region especially since Line157 suggests known QTLs in the neighborhood. This can be in text or supplemental for reference.

Overall suggestion #2- Consider exploring gene enrichment and potential pathways highlighted within the region. 'DAVID Bioinformatic tool' might be useful. Such information can be included as a table would provide further insight into potential modes of action.

Reviewer #2:

The manuscript makes a commendable effort to tackle a crucial issue in soybean-rhizobia symbiosis. The study's design, particularly its focus on identifying novel candidate genes related to symbiotic compatibility in natural conditions, is impressive. This research is significant as it addresses a key aspect of sustainable agriculture by potentially reducing the reliance on chemical fertilizers through enhancing biological nitrogen fixation. Identifying and understanding the genetic basis of symbiotic compatibility in soybeans can lead to the development of soybean varieties with improved efficiency in nitrogen fixation, which is essential for increasing crop yields and soil health. The utilization of a RILs population derived from a cross between 'Peking' and 'Tamahomare' provides for QTL mapping. This approach allows for the identification of specific genomic regions associated with the symbiotic traits. The use of RNA-seq data to identify differentially expressed genes within the QTL regions adds depth to the study by giving a functional context to the genetic findings. However, there are several concerns that need to be addressed before it can be considered for publication:

Main concerns:

1. Experimental Repetition and Validation: The study lacks validation across different environments and years. While it is crucial to repeat the experiments under various environmental conditions and across multiple growing seasons to confirm the stability and applicability of the identified QTLs, if such data is not available, the authors should provide a more detailed experimental design. This detailed design should include strategies to minimize the impact of random factors on the results, ensuring the reliability and environmental adaptability of the findings.

2. Methodological Details: Some methods and results are not described in sufficient detail. For instance, in the RNA-Seq section, while a reference pipeline is provided, more information is needed on sample collection, sampling criteria, inclusion of nodules, and sequencing data specifics. Additionally, a basic presentation of the differential expression analysis results among samples is necessary.

3. Molecular Validation: Although RNA-Seq identified a key gene within the QTL region, validating these findings using a gold standard like qRT-PCR is essential to confirm the differential expression results. This validation step helps to account for potential inaccuracies

inherent in RNA-Seq data.

Minor concerns:

1. Soil Conditions: The manuscript should clarify whether the soil used in the planting experiments inherently contained the two rhizobial species or if they were introduced later. If native soil was used, how did the authors ensure that the soil conditions were as consistent as possible across different samples? This information is crucial for assessing the reliability of the experimental results.

2. Some of the figures lack clarity in their explanations and do not provide detailed descriptions of the markers and symbols used. It is recommended to add detailed annotations below Table 1 to explain terms such as QTL position, LOD score, and phenotypic variance.

3. Figure 1 currently lacks detailed legends and annotations, which may cause confusion for the readers. It is recommended to add legends below or beside the figure to explain the genotypes represented by A and B, as well as the meaning of the PT numbers. Additionally, the percentage data on the right side of the table lacks detailed explanations and should be clarified.

4. Figure S1 does not indicate the LOD threshold line. Adding a LOD threshold line in the figure would help readers identify significant QTL positions. For example, if the threshold is 3, mark and draw a horizontal line on the y-axis. Additionally, the x-axis and y-axis labels may not be sufficiently clear and should be improved.
In Figure S3, it is recommended to use different colors or shapes to distinguish between different types of markers (e.g., GrasDi markers and SSR markers) for a more intuitive differentiation.

5. The heatmap in Figure S4 lacks a necessary legend, making it difficult for readers to intuitively understand the specific fold changes represented by the shades of red and green. It is recommended to include this information.

6. In Figure S5, it is recommended to use different colors or shapes to distinguish between the different parts such as tRNA-Ile, tRNA-Ala, and 23S rRNA. This would provide a more intuitive visualization of the distinct sections.

Future Directions:

1. Functional Validation: While the identification of a candidate gene through QTL mapping and RNA sequencing is noteworthy, functional validation of this gene is crucial. Future studies should consider experiments such as gene knockout or overexpression studies to confirm it roles in symbiotic compatibility. This would significantly enhance the persuasiveness of the study.

Decision Letter Round 2:

March 26, 2025

Prof. Masayoshi Teraishi
Kyoto Daigaku
agriculture
Kitashirakawa Oiwakecho, Sakyo-ku, Kyoto
Kyoto, Japan 606-8502

MSID: 2024-01503R1
MS TITLE: Identification of novel candidate genes associated with the symbiotic compatibility of soybean with rhizobia under natural conditions

Dear Dr. Masayoshi Teraishi:

I am pleased to inform you that your manuscript "Identification of novel candidate genes associated with the symbiotic compatibility of soybean with rhizobia under natural conditions" has been accepted for publication in Plant Direct.

Your article will appear online in the next available issue of Plant Direct. To ensure your article gets published as quickly as possible, please pay attention to the steps detailed below. We have found that most of the delays happen at this stage, especially at the payment stage, so please respond as quickly as possible when prompted.

License Agreement: Once your article has been accepted it will move to Production and undergo admin and file checking - you may receive an email with any queries we have at this stage. When all required items are received by the publisher and queries resolved, the corresponding author will receive an email from Wiley's Author Services system which will ask

them to log on at https://authorservices.wiley.com/bauthor and will present them with the appropriate license for completion. Your article cannot be published until both the signed license agreement and payment of the article fee have been received.

Payment of the Open Access Article Publication Fee: All articles published in Plant Direct are fully open access: immediately and freely available to read, download and share. Plant Direct charges a publication fee to cover the publication costs. The corresponding author for this manuscript should have already received a quote from the payments team (cs-openaccess@wiley.com) with the estimated article publication fee; please email cs-openaccess@wiley.com if this has not been received. The corresponding author should log on to the Wiley Author Services site, where the publication fee can be paid by credit card or an invoice. Pro Forma can also be requested. Payment of the publication charge must be received before the article will be published online.

Proofs: You will have the opportunity to look over your paper once more when you receive the author proofs for your article. The proofs will be with you in approximately two weeks. Please note that, in addition to publishing reviewer comments, the author's responses to review comments will also be published alongside the final version of the paper. If you would not like the author's responses to be published, please contact the editorial office at plantdirect@wiley.com .

Promotion of your article: You can help your research get the attention it deserves! Check out Wiley's free Promotion Guide for best-practice recommendations for promoting your work at www.wileyauthors.com/eeo/guide. And learn more about Wiley Editing Services which offers professional video, design, and writing services to create shareable video abstracts, infographics, conference posters, lay summaries, and research news stories for your research at www.wileyauthors.com/eeo/promotion.

Thank you again for your contribution to Plant Direct. If you have any questions, feel free to contact the editorial office at plantdirect@wiley.com .

Sincerely,

Jonathan Fresnedo-Ramírez
Academic Editor
Gustavo MacIntosh
Editor

Response to Reviewer:

Dear Editors and Reviewers:

Thank you very much for reviewing our manuscript and offering valuable advice. We have addressed your comments with point-by-point responses and revised the manuscript accordingly.

Editor comments:
Please, pay particular attention to the main concerns from Reviewer 2. Plant Direct emphasizes sound scientific studies and ensure the replication of the studies is paramount. qRT-PCR validation would be highly desirable to support the soundness of your study and results.

Reviewer comments:
Reviewer #1:

Line 70 - Briefly mention the predominant soil type or any characterization available of the location where the soil was sampled. Example: (Ultisol, Ph 4.7, OM 2.5%)

Response: Thank you for your suggestion. We have accordingly provided information regarding the soil type used as follows: " (gray lowland soil, pH 6.2, organic matter content 2.3%)" in section 2.1 Plant materials.

Line 71- Was the soil ground after collection? If, yes add it to the description.
Response: Thank you for your suggestion, we have accordingly included the following sentence in the revised manuscript: "Soil samples were ground prior to analysis."

Line 97 - Redundant statement, perhaps use "Twenty-four forward and reverse primers were mixed"
Response: Thank you. We have revised the indicated text as follows: "Twenty-four forward and

reverse primers were mixed to generate 576 primer sets containing 10.0 µM concentrations of each primer.”

Line 110 to 115 - Include the version of the software used. Include what range parameter was considered good quality for the final fastq files.
Response: Thank you for your suggestion. We have accordingly provided details of the software versions and information pertaining to the parameters used.

Line 137 - restructure to avoid starting the sentence with a reference. Perhaps used "Previous reports"

Response: Thank you for pointing out this issue. We have deferred to your advice.

Line 118- Expand GRAS-Di technologies and remove them from Line 142
Response: Thank you for pointing out this issue. We have accordingly defined GRAS-Di as “genotyping by random amplicon sequencing-Direct.”

Line 143- "cM" Centimorgans, verify accordingly through text
Response: Thank you for highlighting this error. We have changed “cm” to “cM.”

Line 178- Add references (Rps genes could work)

Response: We have added reference for the Rps genes (i.e., McHale et al., 2006)

Line 240 to 246- Different font detected, verify accordingly
Response: Thank you for highlighting this inconsistency. We have changed the font in the revised manuscript.

Line 249- Merge with paragraph, space detected.
Response: Thank you for indicating this issue. We have accordingly deleted the space and merged the paragraph.

Table 1 - (3) phenotypic variation symbol missing within the final column of the figure.
Response: Thank you for indicating this omission. We have added“(3)” within the final column.

Figure S1- Add to the description. Example: software from where the figure was generated.

Response: Thank you for your suggestion. We have added relevant information below Figure S1.

Figure S4 - Blurry axis for the genes. Also, there might be image stretching, Make sure it's at least 300DPI.
Response: We apologize for the blurry image. We have enhanced the resolution of the figure.
Table S3- (5) P-value adjusted based on? Bonferroni?
Response: We apologize for this omission. We have accordingly indicated the adjustment method used below Table S3.

Overall suggestion #1- It might be a good idea to include a simple table with colocalizing QTLs to the region especially since Line157 suggests known QTLs in the neighborhood. This can be in text or supplemental for reference.
Response: Thank you for your valued suggestion. We have accordingly provided a further supplementary table (Table S5), listing known QTLs within in Soybase database.

Overall suggestion #2- Consider exploring gene enrichment and potential pathways highlighted within the region. 'DAVID Bioinformatic tool' might be useful. Such information can be included as a table would provide further insight into potential modes of action.
Response: Thank you for this suggestion. We will certainly consider this approach in further studies.

Reviewer #2:

The manuscript makes a commendable effort to tackle a crucial issue in soybean-rhizobia symbiosis. The study's design, particularly its focus on identifying novel candidate genes related to symbiotic compatibility in natural conditions, is impressive. This research is significant as it addresses a key aspect of sustainable agriculture by potentially reducing the reliance on chemical fertilizers through enhancing biological nitrogen fixation. Identifying and understanding the genetic basis of symbiotic compatibility in soybeans can lead to the development of soybean varieties with improved efficiency in nitrogen fixation, which is essential for increasing crop yields and soil health. The utilization of a RILs population derived from a cross between 'Peking' and 'Tamahomare' provides for QTL mapping. This approach allows for the identification of specific genomic regions associated with the symbiotic traits. The use of RNA-seq data to identify differentially expressed genes within the QTL regions adds depth

to the study by giving a functional context to the genetic findings. However, there are several concerns that need to be addressed before it can be considered for publication:

Main concerns:

1. Experimental Repetition and Validation: The study lacks validation across different environments and years. While it is crucial to repeat the experiments under various environmental conditions and across multiple growing seasons to confirm the stability and applicability of the identified QTLs, if such data is not available, the authors should provide a more detailed experimental design. This detailed design should include strategies to minimize the impact of random factors on the results, ensuring the reliability and environmental adaptability of the findings.

Response: Thank you for providing this constructive assessment of our study. Although the experimental design does not include replication, in our previous study (Ramongolalaina et al., 2018), we used three different types of soil (from a paddy field at our university farm and from a soybean field at a distant location from our university) for our nodule research and found that, in all three assessed soil types, the QTLs associated with nodule proportion are located within the same chromosomal region. Therefore, we have been able to establish that the genetic factors regulating the proportion of nodules are consistently identified in the chromosomal region described in the present study.

Moreover, we have re-written parts of the "Materials and Method" section to provide a more detailed description of soybean cultivation. Essentially, we used the same plant incubators under uniform conditions to raise soybean seedlings because we are apprehensive that certain environmental conditions may have an influence on nodulation.

2. Methodological Details: Some methods and results are not described in sufficient detail. For instance, in the RNA-Seq section, while a reference pipeline is provided, more information is needed on sample collection, sampling criteria, inclusion of nodules, and sequencing data specifics. Additionally, a basic presentation of the differential expression analysis results among samples is necessary.

Response: Thank you for highlighting the lack of detail regarding our methodology. We have accordingly provided detailed information in the "Materials and Methods" and "Results" sections of the revised manuscript.

3. Molecular Validation: Although RNA-Seq identified a key gene within the QTL region, validating these findings using a gold standard like qRT-PCR is essential to confirm the differential expression results. This validation step helps to account for potential inaccuracies inherent in RNA-Seq data.

Response: Thank you for your suggestion. Unfortunately, however, the freezer in which our RNA samples were stored broke down earlier this year, and consequently we lost these sample and were thus unable to perform qRT-PCR validation. Moreover, given that the NBS-LRR homologous sequences showed more than 90% similarity, it was difficult to design suitable primers to enable repetition.

Minor concerns:

1. Soil Conditions: The manuscript should clarify whether the soil used in the planting experiments inherently contained the two rhizobial species or if they were introduced later. If native soil was used, how did the authors ensure that the soil conditions were as consistent as possible across different samples? This information is crucial for assessing the reliability of the experimental results.

Response: Thank you for your suggestion. In the revised manuscript, we have included additional information regarding the soil used in this study and emphasized that we used natural uninoculated soil. Although we have performed similar studies over multiple years, the results of experiments are closely comparable.

2. Some of the figures lack clarity in their explanations and do not provide detailed descriptions of the markers and symbols used. It is recommended to add detailed annotations below Table 1 to explain terms such as QTL position, LOD score, and phenotypic variance.

Response: Thank you for your suggestion. As indicated, we have provided the relevant details regarding Table 1, and for other tables and figures.

3. Figure 1 currently lacks detailed legends and annotations, which may cause confusion for the readers. It is recommended to add legends below or beside the figure to explain the genotypes represented by A and B, as well as the meaning of the PT numbers. Additionally, the percentage data on the right side of the table lacks detailed explanations and should be clarified.

Response: Thank you for your suggestion. As indicated in our response to the previous comment, Figure 1 has been supplemented with more detailed information.

4. Figure S1 does not indicate the LOD threshold line. Adding a LOD threshold line in the figure would help readers identify significant QTL positions. For example, if the threshold is 3, mark and draw a horizontal line on the y-axis. Additionally, the x-axis and y-axis labels may not be sufficiently clear and should be improved.
In Figure S3, it is recommended to use different colors or shapes to distinguish between different types of markers (e.g., GrasDi markers and SSR markers) for a more intuitive differentiation.
Response: Thank you for your suggestion. In Figure S1, we have added a horizontal line representing the LOD threshold (3.0) and the axis labels have been suitably enhanced. Furthermore in Figure S3, we have changed the font color of the GRAS-Di markers.

5. The heatmap in Figure S4 lacks a necessary legend, making it difficult for readers to intuitively understand the specific fold changes represented by the shades of red and green. It is recommended to include this information.

Response: We apologize for this omission, and have accordingly provided a relevant legend in the upper right-hand corner of the figure.

6. In Figure S5, it is recommended to use different colors or shapes to distinguish between the different parts such as tRNA-Ile, tRNA-Ala, and 23S rRNA. This would provide a more intuitive visualization of the distinct sections.
Response: Thank you for your suggestion. We have changed these colors.

Future Directions:

1. Functional Validation: While the identification of a candidate gene through QTL mapping and RNA sequencing is noteworthy, functional validation of this gene is crucial. Future studies should consider experiments such as gene knockout or overexpression studies to confirm it roles in symbiotic compatibility. This would significantly enhance the persuasiveness of the study.

Response: Thank you for your suggestion. We appreciate the importance of performing complementary experiments to validate our results. However, prior to undertaking such validation, it will be necessary to assess the copy number variation of these NBS-LRR genes,

given that rust resistance is influenced by a deficiency or duplication of the three tandem NBS-LRR genes (Glyma.18g281500, 281600, and 281700), which includes our candidate gene (Wei et al., 2023). At present, we are yet to examine the chromosomal sequences surrounding the candidate gene in the parent cultivars of the RILs. Nevertheless, we intend to perform complementary experiments, including gene knockout analysis in future.
